# Supplementary material for: Mechanisms governing the pioneering and redistribution capabilities of the non-classical pioneer PU.1
Source: Nat Commun. 2020 Jan 21;11:402. doi: 10.1038/s41467-019-13960-2 (PMC6972792; doi:10.1038/s41467-019-13960-2)
Supplement: Supplementary file 7 — Source data [file 41467_2019_13960_MOESM7_ESM.zip › Source_Data/FigureS2/FigureS2B_MotifScanOutput/KmeansCluster9/homerResults/motif9.similar.html]

motif9

## Information for motif9

T
C
A
G
A
T
G
C
G
A
C
T
A
G
C
T
G
T
A
C
A
G
T
C
C
G
A
T
T
G
A
C
G
A
T
C
T
G
A
C
A
G
T
C
G
A
T
C
  
Reverse Opposite:  

C
T
A
G
T
C
A
G
A
C
T
G
C
T
A
G
A
C
T
G
C
G
T
A
C
T
A
G
C
A
T
G
T
C
G
A
C
T
G
A
T
A
C
G
A
G
T
C
  

|  |  |
| --- | --- |
| p-value: | 1e-44 |
| log p-value: | -1.034e+02 |
| Information Content per bp: | 1.710 |
| Number of Target Sequences with motif | 216.0 |
| Percentage of Target Sequences with motif | 9.49% |
| Number of Background Sequences with motif | 1368.6 |
| Percentage of Background Sequences with motif | 2.97% |
| Average Position of motif in Targets | 100.5 +/- 47.8bp |
| Average Position of motif in Background | 101.6 +/- 67.2bp |
| Strand Bias (log2 ratio + to - strand density) | 0.2 |
| Multiplicity (# of sites on avg that occur together) | 1.02 |
| Motif File: | file (matrix) reverse opposite |

### Similar de novo motifs found

|  |  |  |  |  |  |  |  |
| --- | --- | --- | --- | --- | --- | --- | --- |
| Rank | Match Score | Redundant Motif | P-value | log P-value | % of Targets | % of Background | Motif file |
| 1 | 0.833 | G T A C A G T C A C G T A G T C A G T C A G C T G T A C G A T C A G T C G T A C | 1e-20 | -48.245117 | 6.81% | 2.80% | motif file (matrix) |
| 2 | 0.815 | A T C G A C T G G A T C G A C T G A C T T G A C A G T C A C G T A T G C G T A C T A G C | 1e-20 | -47.601539 | 4.70% | 1.55% | motif file (matrix) |
